# Evaluation of the diagnostic performance of PanbioTM Abbott SARS-CoV-2 rapid antigen test for the detection of COVID-19 from suspects attending ALERT center

**DOI:** 10.1371/journal.pone.0277779

**Published:** 2022-11-22

**Authors:** Wondimu Ashagre, Abay Atnafu, Liya Wassie, Rea Tschopp, Dessalegn Fentahun, Gebeyehu Assefa, Teklu Wegayehu, Biniam Wondale, Andargachew Mulu, Adane Miheret, Kidist Bobosha

**Affiliations:** 1 Arba Minch University, Arba Minch, Ethiopia; 2 Armauer Hansen Research Institute, Addis Ababa, Ethiopia; 3 Swiss Tropical and Public Health Institute, Basel, Switzerland; University of Hail, SAUDI ARABIA

## Abstract

**Background:**

The emergence and rapid spread of coronavirus disease 2019 (COVID-19), a potentially lethal disease caused by severe acute respiratory syndrome coronavirus-2 (SARS-CoV-2), is causing public health issues around the world. In resource-constrained nations, rapid Abbott SARS-CoV-2 antigen test kits are critical for addressing diagnostic gaps in health institutions and community screening. However, there is no evidence or proof of diagnostic performance in Ethiopia. The aim of this study was to compare the performance of PanbioTM Abbott SARS-CoV-2antigen rapid test kit to the gold standard, RT-PCR, in COVID-19 patients with clinical symptoms suggestive of COVID-19.

**Method:**

A prospective, cross-sectional study was conducted between November 2021 and April 2022, on 120 suspected patients recruited from outpatient, emergency, and intensive care units in one of the tertiary hospitals in Ethiopia. Nasopharyngeal swabs were collected from suspected cases and were tested using the Abbott SARS-CoV-2 kit, a rapid diagnostic test (RDT) and compared to the reference standard RT-PCR.

**Result:**

The sensitivity and specificity of the RDT were 74.2% and 100%, respectively. A total of 62 samples (51.6%) were RT-PCR positive. Of these, 46 were Ag-RDT positive. Sensitivity among symptomatic patients was 79.4% (95% CI 68.3–90). The Abbot RDT and RT-PCR had a Kappa value of agreement of 0.735 (p < 0.001). These values were acceptable when compared to the WHO’s suggested thresholds.

**Conclusion:**

The finding from this study support the use of the Abbot RDT as a diagnostic tool in COVID-19 suspects, mainly in those with higher viral loads.

## Introduction

COVID-19 is a disease caused by severe acute respiratory syndrome corona virus 2 (SARS-CoV-2), an RNA virus that causes severe acute respiratory syndrome [1]. COVID-19 was originally discovered in individuals with viral pneumonia symptoms in Wuhan, China, in December 2019. As the start of the study on October 18, 2021, globally, there were about 241,411,380 million confirmed cases and 4,912,112 deaths, where with almost 44 million cases and 722,690 deaths, the United States of America was the most afflicted country. In Ethiopia, as of October 17, 2021, there were 359,881 confirmed cases and 6,258 COVID-19 death report [2].

Patients over the age of 65 and those who have pre-existing illnesses (such as hypertension, heart disease, lung disease, cancer, or diabetes) have been identified as possible risk groups for severe disease and mortality [3]. Health Care Workers’ lack of understanding of the disease can lead to delayed detection, identification, and treatment, resulting in infection transmission [4].

The diagnostic tool utilized in the global surveillance for the detection of COVID-19 involves Nucleic acid amplification tests (NAATs), mainly reverse-transcription polymerase chain reaction (RT-PCR) assays [5]. Currently, a number of NAATs are commercially available for usage; however, the testing capacity of NAATs is insufficient to deal with the COVID-19 pandemic, due to the need for qualified staff, the cost of the test, and specialized viral detection instrumentation [6].

Alternatively various manufacturers have developed SARS-CoV-2 rapid point-of-care antigen tests, providing an advantage over NAATs in that they are rapid, cheaper, and do not require specialized equipment or skilled personnel but they are less sensitive and will miss low viral load [7]. However, its diagnostic performance has not been evaluated in the context of Ethiopia. There is therefore a need to evaluate the performance of antigen testing for diagnosis of COVID-19 [8]. It is thought to deliver early detection of COVID-19 which is critical for optimal disease management and interventions [9].

The World Health Organization (WHO) advises utilizing rapid antigen test kits with a sensitivity of at least 80% and specificity of 97% as compared to the gold standard RT-PCR [2]. Antigen point-of-care tests are currently being employed in several countries to help elevate the diagnostic capacity together with RT PCR [10]. However, in some instances, the test has been criticized for having lesser clinical sensitivity than NAATs [11]. The Abbott COVID-19 rapid antigen testing kit has been utilized in many countries and is currently being distributed in Ethiopia [12].

In this study, we aimed to evaluate the diagnostic performance of the PanbioTM Abbott SARS-CoV-2 antigen test for the detection of COVID-19 as compared to the Gold standard RT-PCR, aiming at distribution and use in all health-care settings.

## Materials and methods

### Study setting

This study is a prospective cross sectional study which enrolled a total of 120 COVID-19 suspected patients attending the All African Leprosy Research and Training (ALERT) Hospital, Addis Ababa, Ethiopia from November 2021 to April 2022. Ethical approval was obtained from AHRI/ALERT Ethics Review Committee. A written informed consent was obtained from each study participants and for those participants whose age were below 18, a parental/guardian consent and child assent was obtained before enrollment into the study. RT-PCR was used as a gold standard to analyze the sensitivity, specificity, positive and negative predictive value of PanbioTM Abbot RDT-Ag test at a Ct cut-off value of ≤40 [12]. The sample size was determined by (https://www.openepi.com/SampleSize/SSCohort.htm). We used to collect 120 samples due to budget and time constraints. The population correction was used. The calculated sample sizes were multiplied by the design effect.

### Data analysis

Data entered and analyzed by SPSS version 23, statistical software. Ag-RDT and RT-PCR result with the corresponding Ct value were collected for all cases. The RDT performance was assessed using sensitivity, specificity, positive predictive value, negative predictive value, and respective 95% Confidence interval. The agreement between RT-PCR a significance value of 0.05 in statistic tests considered (P-values <0.05) [13].

### Sample collection

Nasopharyngeal swabs specimen were collected using viral transport medium (VTM). For the rapid antigen test, a separate swab was collected without VTM, both swab samples were collected in parallel, according to WHO and the national standard operating procedure (SOP) with strict biosafety measures. The nasopharyngeal samples were collected in the morning hour by trained nurses and laboratory technologists from eligible participants attending ALERT hospital, Addis Ababa [14, 15]. Both RDT and RT PCR testing were performed with an hour of swab sample collection at AHRI COVID laboratory.

### Laboratory procedures

#### Rapid Abbott COVID-19Ag test

Five drops of sample buffer were transferred into a small conical tube, then a nasopharyngeal swab collected from each participant was immersed into a tube containing sample buffer supplied with the test kit (Abbott, Panbio, COVID 19 Ag Rapid test device, Jena, Germany), LOT 41ADG095A following five minutes incubation, five drops of mixture were added into a sample well. The kit was left for 15 minutes at room temperature before reporting result as negative. The test was considered positive if two bands was appeared both in the sample and control area [16, 17].

#### Real time PCR

Nasopharyngeal swab samples were used for RNA extraction. Swabs were put into catalogue– 2020060025 VTM tubes with sample preservative fluid after sampling and vortexed vigorously for one minute. 300μL of soaking solution were then placed into a 1.5mL nuclease-free centrifuge tube for use. The major open reading frames ORF1ab gene as domain target, and we followed the manufacturers’ manual (MagaBio Plus Virus RNA Purification Kit Ⅱ, China). The cutoff point for a negative result is 40 cycles. If the virus is detected between 38 and 40 cycles, we call this an indeterminate or inconclusive result. All inconclusive results are considered probable (likely) cases for public health reporting.

The detection kit Beijing Genomics Institute (BGI, Real Time Fluorescent RT-PCR Kit, China) used in this study contains two vials: a reaction and an enzyme mix. The master mix was prepared by mixing 18.5 μl of SARS-COV-2 reaction mix with 1.5 μl of SARS-COV-2 Enzyme mix. After thoroughly mixing, 10 μl of the master mix was added to each well in the reaction plate, which was later mixed with 10μl of the purified RNA. The plate was then placed in to a thermo-cycler (Bio Rad, CFX96, Singapore) for amplification reaction. The PCR protocol used the following: one cycle at 50°C for 20minutes, followed by one cycle at 95°C for 10minutes, and 40 cycles involving 95°C for 15 seconds and 60°C for 30 seconds.

## Results

### Demographic characteristics

A total of 120 participants were enrolled in this study. The mean age of the participants was 35 years with SD of 13.3. 83/120 (69.2%) and 37 (30.8%) were females and males respectively. The majority of the participants (99%) were urban dwellers. Most of the participants (N = 108 had symptoms of COVID-19. Nearly half (N = 59; 49.2%) had received a first-round AstraZeneca vaccine. Among them 30 (50.8%) and, 23 (38.9%) were positive by RT-PCR and Abbot RDT-Ag tests respectively (Table 1).

**Table 1:** Socio-demographic characteristics of study participants (N = 120).

Variables		Total	RT PCR		RDT Ag	
			Positive	Negative	Positive	Negative
Age group	<18	7	3	4	2	5
	19–40	86	49	37	34	52
	41–60	18	8	10	8	10
	>61	9	2	7	2	7
Gender	Male	37	16	21	14	23
	Female	83	46	37	32	51
Occupation	Governmental	98	54	44	40	58
	Self employed	4	3	1	3	1
	Student	7	3	4	2	5
	Unemployed	6	2	4	1	5
	Retired	4	0	4	0	4
	Other	1	0	1	0	1
Vaccine status	Vaccinated	59	30	29	23	36
	Non vaccinated	61	32	29	23	38
Symptom	Yes	108	60	48	45	63
	No	12	2	10	1	11
Cough	Yes	86	45	41	35	52
	No	34	17	17	11	23
Fever	Yes	48	30	18	25	23
	No	72	32	40	21	51
Shortness of breath	Yes	17	7	10	6	11
	No	103	55	48	40	63
Sore throat	Yes	46	30	16	22	24
	No	74	32	42	24	50
Headache	Yes	62	43	19	36	26
	No	58	19	39	10	48
Easy fatigue	Yes	54	38	16	31	23
	No	66	24	42	15	51
Underlying condition	No	112	59	53	43	69
	Asthma	1	0	1	0	1
	Chronic cardiac disease	2	1	1	1	1
	DM & Hypertension	1	1	0	1	0
	Hyper tension	3	1	2	1	2
	HIV	1	0	1	0	1
Contact with COVID-19 patient	No	65	22	43	15	50
	Work place	52	38	14	30	22
	House hold	3	2	1	1	2

Abbot RDT-Ag test detected 46/120 (38.3%) and 74/120 (61.7%) cases as positive and negative respectively. The level of agreement with the gold standard RT-PCR was 0.735 which is said to be good. The RDT- Ag test had an excellent specificity (100%), while the sensitivity decreased to 74.2% (Table 2).

**Table 2:** Detection rate and diagnostic performance of Abbot RDT Ag test against RT-PCR.

Abbot RDT-Ag test	RT-PCR		Total	P value	Kappa value
	Positive	Negative			
Positive	46	0	46	<0.001	0.735
Negative	16	58	74		
Total	62	58	120		
Diagnostic performance	Sensitivity	74.2% (95% CI: 63.3% - 85%)			
	Specificity	100% (95% CI: 100%)			
	PPV	100% (95% CI: 100%)			
	NPV	78.4% (95% CI: 69% - 87.7%)			

In this study, the RDT test did not miss those positive cases with a Ct value of less than 24. However for a positive case with a Ct value of higher than 24, the rapid test missed the majority of the cases, For a Ct of 32 and higher, the Ct value cut-off then is 40, the rapid test detected only 1/12 (8%) of the positive cases as shown in(Table 3).

**Table 3:** Detection rate of Abbot RDT-Ag test as compared with Ct values (N = 120).

Ct value	RDT Ag		Total
	Positive	Negative	
N/A	0	57	57
Total	46	74	120
**Ct value**	**Sensitivity**		
<27.1	97.40% (95% CI: 92.3–100)		
	**Specificity**		
	100% (95% CI: 100)		
>27.2	**Sensitivity**		
	36% (95% CI: 17.1–54.2)		
	**Specificity**		
	64% (95% CI: 33.5–82.1)		

*N/A (Ct value >40)*.

The majority of the participants (N = 108 (90%) were symptomatic. Of these, 61(56.5%) had a Ct value of below 39 (P-value = 0.045) as shown in (Table 4).

**Table 4:** Comparison of various groups of Ct values with vaccination status and symptoms of the participants.

Ct value	Frequency	Vaccinated		P Value	Symptom		P Value
		Yes	No		Yes	No	
17.1–23.1	18	6	12	0.77	17	1	0.045
23.2–29.2	28	16	12		28	0	
29.3–35.3	14	8	6		13	1	
35.4–41.4	3	0	3		3	0	
**N/A**	63	30	33		61	2	
**Total**	120	59	61		108	12	

## Discussion

The Abbot RDT Ag test detected 46 (38.3%) and 74 (61.7%) cases as positive and negative, respectively and PPV was 100% while NPV was 78.4%. The level of agreement with the gold standard RT-PCR was 0.735, which is considered significant [13]. The RDT-Ag test showed to have an excellent specificity (100%), while the sensitivity decreased to 74.2%. This finding is similar to the study conducted in Mallorca, Spain on PanbioTM abbot Ag-RDT kit, which demonstrated a sensitivity of 79% and a specificity of 100% [11].

Another study conducted in Mozambique during the peak transmission period found a considerably lower sensitivity of 41.3% and a specificity of 98.2%, as well as a PPV of 93.3% and NPV of 52.9%. The study also showed a level of agreement of 0.766 that was similar to our study [12]. A similar study in Uganda also demonstrated a sensitivity of 70% (95% CI: 60–79), a finding which was slightly similar with this study [18].

In Uganda, one study compared the performance of seven Antigen Rapid Diagnostic Tests (RDTs) for the diagnosis of SARS-CoV-2 virus infection. The RDTs included the COVID-19 Ag Respi-Strip, BIOCREDIT COVID-19 Ag, MEDsan1 SARS-CoV-2 Antigen Rapid test, PCL COVID19 Ag Rapid FIA, PanbioTM COVID-19 Ag Rapid test, Novegent COVID-19 Antigen Rapid test kit. Only the PanbioTM COVID-19 Ag Rapid Test meets the WHO performance requirements of 80% sensitivity and 97% specificity at qRT-PCR Ct levels of 29, and our RDT test results are comparable with this study [19].

There was also a clear correlation between the PanbioTM Abbot COVID 19 Ag RDT result and Ct value of RT-PCR [20]. As we determined by the Ct value using RT-PCR; samples with a Ct value of < 24.6 were all positive and samples with a Ct value of >39 were negative with RDT. Similar results were found in Mozambique during in high transmission period, when the Ct value <25 sensitivity of 52.7%, when Ct > 25 sensitivity of 11.9% [12].

In our study, 108 out of 120 were symptomatic. 19 of the symptomatic suspects had been vaccinated with a Ct value of < 27.1, whereas the remaining 40 were immunized with a Ct value > 27.2. Among asymptomatic 12 patients, two were positive with RT-PCR but one was missed with the RDT-Ag; detection of the disease and level of infectiousness are critical for suspected individuals, and if infection is still being transmitted, the RDT-Ag may miss some positive suspects with high Ct values, which is consistent with previous results and likely reflects lower virus levels in those patients. Another study reported that overall sensitivity was 30.4%, but that patients with symptoms had a higher sensitivity of 52.9% [16]. Furthermore, in a different study, the total sensitivity was 65.3% (95% confidence interval: 56.8–73.1) in symptomatic cases and 44.4% (95% confidence interval: 24.4–65.1) in asymptomatic cases [21]. The findings suggest that asymptomatic and pre-symptomatic persons do represent a source of potentially transmissible virus [22].

When compared to the WHO’s indicated standards, the sensitivity was slightly lower than the specified standard, but the specificity was acceptable. WHO advises employing kits with a sensitivity of 80% and a specificity of higher than 97% in its preliminary guidance on using SARS-CoV-2 rapid antigen assays [2, 23]. According to the manufacturer, the stated Abbott SARS-CoV-2 kit sensitivity and specificity was 71.4% and 99.8%, respectively. The Abbott SARS-CoV-2 antigen kit performed well based on WHO and manufacturer-specified sensitivity and specificity.

The availability of RT-PCR testing capacity is limited to a few specialized centers, resulting in significant testing delays. Despite the fact that the new Abbott COVID-19 RDT-Ag kit has slightly reduced sensitivity, we are employing it in a resource-constrained setting because it was simple to use in health facilities and the field and required little training. For health-care workers and patients it would be useful for early quarantining contact tracing, and hospitalization for those who needed it. This test can be distributed to remote areas that have limited availability/access to RT-PCR testing and high patient numbers, hence allowing for rapid test results and short turnaround times [17, 24].

Diagnostic tests with a high PPV should be used when there is a high rate of disease transmission. This gives clinicians and public health officials more confidence in their decisions. In our study, the PPVs were detected in 100%, especially those experiencing symptoms. Thus, during a high transmission period in our setting, at least 90% of those showing positive on RDT-Ag were correctly identified and confirmed using RT-PCR-as SARS-CoV-2 infected [25, 26].

However for a positive case with a higher Ct value, the rapid test missed the majority of the cases, detecting only 1 out of 8 positive cases when the Ct was 32 and higher. RT-PCR testing should be offered if clinical suspicion is high because individuals with low viral load (high Ct values on RT-PCR) have poorer sensitivity [27]. Users should follow the manufacturer’s testing procedures to avoid potentially misleading false-negative or false-positive results [28].

To the best of our knowledge, this is the first study in Ethiopia investigating the performance of COVID-19 Abbot RDT-Ag kit. This test was shown to be useful in tackling the challenges to prevent the spread of SARS-CoV-2 across the community. However, users should be mindful of the RDT kits’ limitations. Studies have found that screening asymptomatic people has a low sensitivity [29].

## Conclusion

While the PanbioTM rapid antigen test for SARS-CoV-2 has a high specificity, it has a relatively low and heterogeneous sensitivity in real life depending on the onset of symptoms and viral load. A person testing negative on the RDT-Ag with suggestive COVID-19 symptoms during a time of community transmission of SARS-CoV-2 needs confirmation with RT-PCR, keeping in mind that sensitivity decreases dramatically after the first week of symptoms. Abbott SARS-CoV2 rapid antigen test can be valuable screening and diagnostic test for COVID-19. The kit nearly met the WHO cut-off standards, when compared to the gold standard for detecting COVID-19. The Abbott quick antigen test is easy to perform, to transport and store and does not require training/skilled laboratory professional, hence it can be used for rapid screening, of patients in Ethiopian health care settings.

Early detection and isolation of cases are essential to slow transmission, and provide timely clinical therapy to patients who are infected and safeguarding health system operations by triaging at admissions.

## Supporting information

S1 Dataset (XLSX)

## References

[1] Sharma A, Tiwari S, Deb MK, Marty JL. Severe acute respiratory syndrome coronavirus-2 (SARS-CoV-2): a global pandemic and treatment strategies. Int J Antimicrob Agents. 2020;56(2):106054–. doi: 10.1016/j.ijantimicag.2020.106054 . Epub 06/10. 32534188 PMC7286265

[2] WHO, Recommendations for national SARS-CoV-2 testing strategies and diagnostic capacities: interim guidance. 2021; (https://covid19.who.int/region/amro/country/us). (https://covid19.who.int/region/afro/country/et).

[3] Flaherty G.T., Hession P., Liew C.H. et al. COVID-19 in adult patients with pre-existing chronic cardiac, respiratory and metabolic disease: a critical literature review with clinical recommendations. *Trop Dis Travel Med Vaccines* 6, 16 (2020). doi: 10.1186/s40794-020-00118-y 32868984 PMC7453673

[4] Olum R, Chekwech G, Wekha G, Nassozi DR, Bongomin F. Coronavirus Disease-2019: Knowledge, Attitude, and Practices of Health Care Workers at Makerere University Teaching Hospitals, Uganda. Frontiers in Public Health. 2020; 2020-April-30;8. 10.3389/fpubh.2020.00181 PMC7204940 32426320

[5] Dickson EM, Zambon M, Pebody R, de Lusignan S, Elliot AJ, Ellis J, et al. (2020). Do point-of-care tests (POCTs) offer a new paradigm for the management of patients with influenza? Euro Surveill; 25(44)2020; 11. 10.2807/1560-7917.ES.2020.25.44.1900420 PMC7645971 33153522

[6] Somborac Bačura A, Dorotić M, Grošić L, Džimbeg M, Dodig S. Current status of the lateral flow immunoassay for the detection of SARS-CoV-2 in nasopharyngeal swabs. Biochemia medica. 2021 Jun 15;31(2):020601. doi: 10.11613/BM.2021.020601 . Pubmed Central PMCID: PMC8183114. Epub 2021/06/19. 34140830 PMC8183114

[7] Fletcher JJ, Feucht EC, Hahn PY, McGoff TN, Dehart DJ, El Mortada ME, et al. Healthcare-acquired coronavirus disease 2019 (COVID-19) is less symptomatic than community-acquired disease among healthcare workers. Infection control and hospital epidemiology. 2021 Apr 15:1–7. doi: 10.1017/ice.2021.167 . Pubmed Central PMCID: PMC8485008. Epub 2021/04/16. 33853694 PMC8485008

[8] Kahn M, Schuierer L, Bartenschlager C, Zellmer S, Frey R, Freitag M, et al. Performance of antigen testing for diagnosis of COVID-19: a direct comparison of a lateral flow device to nucleic acid amplification based tests. BMC Infectious Diseases. 2021 2021/08/10;21(1):798. doi: 10.1186/s12879-021-06524-7 34376187 PMC8354301

[9] Hayer J, Kasapic D, Zemmrich C,. Real-world clinical performance of commercial SARS-CoV-2 rapid antigen tests in suspected COVID-19: A systematic meta-analysis of available data. International Journal of Infectious Diseases. 2021; 108, 592–602. 34015523 10.1016/j.ijid.2021.05.029 PMC8127520

[10] Torres I, Poujois S, Albert E, Colomina J, Navarro D. Evaluation of a rapid antigen test (Panbio™ COVID-19 Ag rapid test device) for SARS-CoV-2 detection in asymptomatic close contacts of COVID-19 patients. Clin Microbiol Infect. 2021;27(4):636.e1–.e4. doi: 10.1016/j.cmi.2020.12.022 . Epub 01/06. 33421573 PMC7833843

[11] Bulilete O, Lorente P, Leiva A, Carandell E, Oliver AR, Rojo E, et al. Panbio™ rapid antigen test for SARS-CoV-2 has acceptable accuracy in symptomatic patients in primary health care. The Journal of Infection. 2021;82:391–8. doi: 10.1016/j.jinf.2021.02.014 33592253 PMC7881288

[12] Sitoe N, Sambo J, Nguenha N, Chilaule J, Chelene I, Loquiha O, et al. Performance Evaluation of the STANDARD(TM) Q COVID-19 and Panbio(TM) COVID-19 Antigen Tests in Detecting SARS-CoV-2 during High Transmission Period in Mozambique. Diagnostics (Basel, Switzerland). 2022;12(2):475. doi: 10.3390/diagnostics12020475 . 35204566 PMC8871422

[13] McHugh ML. Interrater reliability: the kappa statistic. Biochemia medica. 2012;22(3):276–82. . 23092060 PMC3900052

[14] Ngwewondo A, Nkengazong L, Ambe LA, Ebogo JT, Mba FM, Goni HO, et al. Knowledge, attitudes, practices of/towards COVID 19 preventive measures and symptoms: A cross-sectional study during the exponential rise of the outbreak in Cameroon. PLoS Negl Trop Dis.;2020;14(9):e0008700. doi: 10.1371/journal.pntd.0008700 32886678 PMC7497983

[15] Wang H, Liu Q, Hu J, Zhou M, Yu M-Q, Li K-Y, et al. Nasopharyngeal Swabs Are More Sensitive Than Oropharyngeal Swabs for COVID-19 Diagnosis and Monitoring the SARS-CoV-2 Load. Frontiers in Medicine. 2020 2020-June-18;7. doi: 10.3389/fmed.2020.00334 32626720 PMC7314917

[16] Mboma O, Rieke E, Ahmad-Nejad P, Wirth S, Aydin M. Diagnostic Performance of SARS-CoV-2 Rapid Antigen Test in a Large, German Cohort. Children. 2021;8. 34438573 10.3390/children8080682 PMC8394520

[17] Tech A. Abbott’s FreeStyle® Libre 2 iCGM Cleared in U.S. for Adults and Children with Diabetes, Achieving Highest Level of Accuracy and Performance Standards. CISION PR newswire. 2020.

[18] Nalumansi A, Lutalo T, Kayiwa J, Watera C, Balinandi S, Kiconco J, et al. Field evaluation of the performance of a SARS-CoV-2 antigen rapid diagnostic test in Uganda using nasopharyngeal samples. Int J Infect Dis. 2021 Mar;104:282–286. doi: 10.1016/j.ijid.2020.10.073 Epub 2020 Oct 30. ; PMCID: PMC7836828. 33130198 PMC7836828

[19] Bwogi J, Lutalo T, Tushabe P, Bukenya H, Eliku JP, Ssewanyana I, et al. Field evaluation of the performance of seven Antigen Rapid diagnostic tests for the diagnosis of SARsCoV-2 virus infection in Uganda. PLoS ONE (2022);17(5): e0265334. doi: 10.1371/journal.pone.0265334 35536792 PMC9089886

[20] Al Bayat S, Mundodan J, Hasnain S, Sallam M, Khogali H, Ali D, et al. Can the cycle threshold (Ct) value of RT-PCR test for SARS CoV2 predict infectivity among close contacts? Journal of infection and public health. 2021 Sep;14(9):1201–5. doi: 10.1016/j.jiph.2021.08.013 . Pubmed Central PMCID: PMC8362640. Epub 2021/08/21. 34416598 PMC8362640

[21] Jegerlehner S, Suter-Riniker F, Jent P, Bittel P, Nagler M. Diagnostic accuracy of a SARS-CoV-2 rapid antigen test in real-life clinical settings. International journal of infectious diseases: IJID: official publication of the International Society for Infectious Diseases. 2021 Aug;109:118–22. doi: 10.1016/j.ijid.2021.07.010 . Pubmed Central PMCID: PMC8260496. Epub 2021/07/10. eng. 34242764 PMC8260496

[22] Singanayagam A, Patel M, Charlett A, Lopez Bernal J, Saliba V, Ellis J, et al. Duration of infectiousness and correlation with RT-PCR cycle threshold values in cases of COVID-19, England, January to May 2020. Euro surveillance: bulletin Europeen sur les maladies transmissibles = European communicable disease bulletin. 2020 Aug;25(32). doi: 10.2807/1560-7917.ES.2020.25.32.2001483 . Pubmed Central PMCID: PMC7427302. Epub 2020/08/15. 32794447 PMC7427302

[23] Crozier A, Rajan S, Buchan I, McKee M. Put to the test: use of rapid testing technologies for covid-19. BMJ (Clinical research ed). 2021 Feb 3;372:n208. doi: 10.1136/bmj.n208 . Epub 2021/02/05. 33536228

[24] Gitman M.R.; Shaban M.V.; Paniz-Mondolfi A.E.; Sordillo E.M.,. Laboratory Diagnosis of SARS-CoV-2 Pneumonia. Diagnostics, 2021;11, 1270. doi: 10.3390/diagnostics11071270 34359353 PMC8306256

[25] Jo SJ, Shin S, Kim J, Lee S, Lee J. Evaluation of the clinical performance of a magnetic force-assisted electrochemical immunoassay for the detection of SARS- CoV-2 antigens. PLoS ONE. 2021;16(10): e0258394. doi: 10.1371/journal.pone.0258394 34618868 PMC8496795

[26] Alqahtani M, Abdulrahman A, Mustafa F, Alawadhi AI, Alalawi B, Mallah SI. Evaluation of Rapid Antigen Tests Using Nasal Samples to Diagnose SARS-CoV-2 in Symptomatic Patients. Front Public Health. 2022 Jan 14;9:728969. doi: 10.3389/fpubh.2021.728969 ; PMCID: PMC8795669. 35096725 PMC8795669

[27] Gietema HA, Zelis N, Nobel JM, et al. CT in relation to RT-PCR in diagnosing COVID-19 in The Netherlands: A prospective study. PLoS One. 2020;15(7):e0235844. Published 2020 Jul 9. doi: 10.1371/journal.pone.0235844 32645053 PMC7347219

[28] Tahamtan A, Ardebili A. Real-time RT-PCR in COVID-19 detection: issues affecting the results. Expert review of molecular diagnostics. 2020 May;20(5):453–4. doi: 10.1080/14737159.2020.1757437 . Pubmed Central PMCID: PMC7189409. Epub 2020/04/17. 32297805 PMC7189409

[29] Sicilia P, Castro G, Fantilli AC, Gierotto R, López L, Barbás MG, et al. Rapid screening of SARS-CoV-2 infection: Good performance of nasopharyngeal and Nasal Mid-Turbinate swab for antigen detection among symptomatic and asymptomatic individuals. PLOS ONE. 2022;17(4):e0266375. doi: 10.1371/journal.pone.0266375 35363814 PMC8986327

