# Correction: Evaluation of the diagnostic performance of PanbioTM Abbott SARS-CoV-2 rapid antigen test for the detection of COVID-19 from suspects attending ALERT center

**DOI:** 10.1371/journal.pone.0307338

**Published:** 2024-07-12

**Authors:** Wondimu Ashagre, Abay Atnafu, Liya Wassie, Rea Tschopp, Dessalegn Fentahun, Gebeyehu Assefa, Teklu Wegayehu, Biniam Wondale, Andargachew Mulu, Adane Mihret, Kidist Bobosha

The tenth author’s name is spelled incorrectly. The correct name is: Adane Mihret.
